# Aggregation Dynamics of Colloidal Particles in Tin Perovskite Crystalline Film Formation

**DOI:** 10.1021/acsenergylett.5c02847

**Published:** 2025-10-28

**Authors:** Davide Amoroso, Giuseppe Nasti, Massimiliano Maria Villone, Tim Kodalle, Carolin Maria Sutter-Fella, Pier Luca Maffettone, Antonio Abate

**Affiliations:** † Department of Chemical, Materials and Manufacturing Engineering, University of Naples Federico II, Piazzale Vincenzo Tecchio, 80, 80125, Naples, Italy; ‡ Enea Research Center Portici, Piazzale Enrico Fermi 1, 80055 Portici, Italy; § Molecular Foundry, 1666 Lawrence Berkeley National Laboratory, Berkeley, California 94720, United States

## Abstract

We present an approach to understanding the crystallization of tin-based perovskite films for photovoltaic applications, starting from precursor suspensions processed via spin-coating. By integrating colloidal theory with the fluid dynamics of suspensions, this approach elucidates the influence of both chemical variables and process parameters on the crystallization pathways of perovskite suspensions and the resulting microstructural features of the solid films. Specifically, the incorporation of SnCl_2_ as an additive was found to accelerate crystallization, whereas tBP induces a slowdown of the process leading, however, to a marked improvement in film uniformity and microstructural quality.

## Graphical abstract

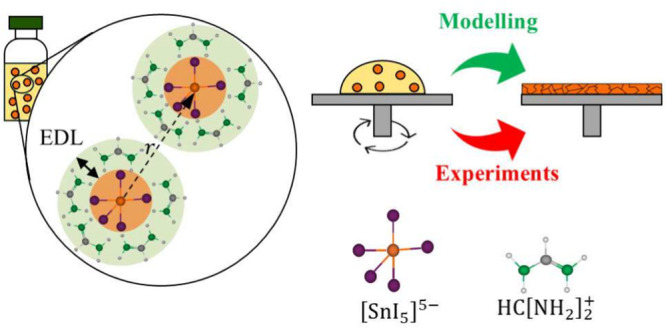

Perovskite materials, characterized by the ABX_3_ crystalline structure (A organic/inorganic cation, B metal cation, X halide anion), attract significant attention in the photovoltaic industry due to their high efficiency and low production costs.^1–3^ However, their industrial adoption is hindered by difficulties in controlling the microstructure of the perovskite films, which is critical for solar cell performance and stability,^4–6^ and concerns about the environmental impact of large-scale adoption of lead organic salts.^7^ For this reason, the scientific community is exploring tin-based perovskites as an environmentally safer alternative to lead-based ones, offering a promising future for this solar cell technology. In addition, tin-based perovskites can even reach theoretically higher efficiencies than their lead-based counterparts^8^ and be used for tandem solar cells.^9^

Controlling the microstructure of perovskites is highly challenging, as it requires precise tuning of many variables, including the chemical and physical properties of the perovskite precursor solutions^10,11^ and the parameters associated with the manufacturing processes.^3,12,13^ The challenges associated with tin-based perovskites are even more severe because tin favors rapid crystallization and is more prone to oxidation.^14,15^ Therefore, a purely parametric approach, in which properties such as concentration, additive-to-perovskite ratio, and process variables are optimized through experimental iteration, proves to be a highly complex and inefficient way to understand the full range of crystallization mechanisms involved in perovskite film formation.

This letter introduces a pioneering approach to studying the crystallization mechanisms of tin-based perovskite films during spin-coating, rooted in colloidal theory^16^ and suspension fluid dynamics. Our core contribution is the demonstration that specific additives can be engineered to actively alter the fundamental dynamics of crystal formation, thereby dictating film microstructure. We initiate our study by characterizing perovskite suspensions as colloidal systems under realistic spin-coating flow. Subsequently, we detail our experimental setup and provide a rigorous analysis of the crystallization process, culminating in a clear exposition of how additives critically influence the resulting film microstructure.

In recent years, it has been recognized that those we usually refer to as ‘perovskite precursor solutions’ are colloidal suspensions rather than chemical solutions.^17–19^ As illustrated in the left part of Figure 1, dissolving precursor salts, such as formamidinium iodide, FAI, and tin iodide, SnI_2_, in solvent results in a suspension of colloidal particles, where each colloid is an iodostannate ion, ${\left[{\mathrm{SnI}}_{5}\right]}^{5-}$ , having five attachment sites, and the electronic double layer (EDL) is composed of organic perovskite cations (specifically, formamidinium ions, $HC{\left[NH_{2}\right]}_{2}^{+}$ (assumed the same configuration as in Flatken et al.^18^). The right part of Figure 1 shows a representative colloidal interaction potential as described by the well-known Derjaguin, Landau, Verwey, and Overbeek (DLVO) theory.^20^ The potential profile has a maximum that represents the energy barrier, i.e., *U*_m_, that must be overcome for colloidal aggregation to occur.^16,21^ A higher maximum indicates stronger repulsive forces, reducing the likelihood of colloid aggregation, whereas a lower maximum suggests weaker repulsive forces, thus a greater tendency to aggregation.^22^ For the purposes of our discussion, we neglect the possible presence of long-distance secondary minimum.

**Figure 1:**
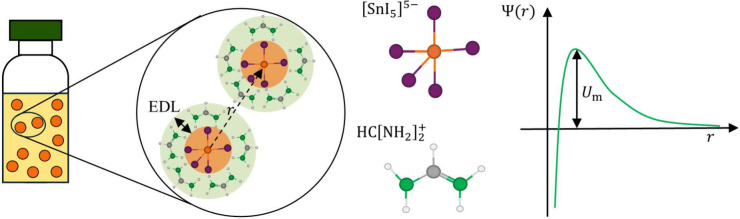
Left: Schematic description of a FASnI_3_ perovskite colloidal suspension. The core of each particle (orange regions) is the iodostannate ion, whereas the EDL (green regions) is composed of formamidinium organic cations. Right: Aggregation barrier as a function of the distance from the core of a particle (qualitative diagram).

When a colloidal suspension is subjected to flow, its properties can be altered depending on the type of the applied flow.^23–25^ Zaccone et al.^25^ showed that, in a simple shear flow, the rate of colloidal aggregation is influenced not only by the chemical and physical properties of the suspension but also by the Péclet number, Pe, which measures the relative importance of convective and thermal motion of the colloids.^26^

On a laboratory scale, spin-coating is the most commonly employed method for fabricating perovskite-based solar cells due to its simplicity and cost-effectiveness.^3^ This process has been widely used to produce various coatings since the 1950s.^27^ Given a fluid rotating on a circular substrate, the characteristic shear rate can be defined^27,28^ as $\mathrm{\gamma ̇}=\rho \omega^{2}Rh_{0}/\mu$ , where ρ is the fluid density, ω is the angular velocity, *R* is the substrate radius, *h*_0_ is the film thickness at the beginning of the process, and μ is the fluid viscosity. For concentrated colloidal systems, such as those investigated in this study, Zaccone’s theory must be modified to account for interparticle interactions. One suitable framework for addressing this complexity is the ‘effective medium’ approach,^29^ in which the solvent is replaced by an effective fluid whose properties reflect the collective behavior of the suspension. Assuming that the Stokes–Einstein relation and the ‘effective medium’ approach hold, the concentration-dependent Péclet number characterizing the spin-coating of a colloidal suspension can be written as

1 $$Pe\left(\phi \right)=\frac{\mathrm{\gamma ̇}a^{2}}{D\left(\phi \right)}=\frac{3\pi \mu_{r}\left(\phi \right)\rho \omega^{2}Rh_{0}a^{3}}{k_{B}T}$$

where ϕ is the volume fraction of the suspended particles, *a* is the colloidal particle radius, *D*(ϕ) is the diffusion coefficient of the particles in the surrounding fluid (depending on concentration), μ_r_ is the relative viscosity of the suspension (also depending on concentration), *k*_B_ is the Boltzmann constant, and *T* is the absolute temperature. Assuming that, under the given process conditions, the material remains uniformly distributed throughout the evaporation phase of spin-coating, the progressive loss of solvent results in an increase in the solute volume fraction. This, in turn, leads to a corresponding rise in the relative viscosity of the suspension.^21,30^ Therefore, as all the other parameters are kept constant, Pe increases while the spin-coating operation continues. Based on the approach adopted by Zaccone et al.,^25^ the concentration-dependent colloidal aggregation rate, *k*, can be estimated as

2 $$k\left(\phi \right)\approx f\left(\mathrm{\gamma ̇}\right)\exp\left(-\frac{U_{m}}{k_{B}T}+\alpha \mathrm{Pe}\left(\phi \right)\right)$$

where f(γ̇) is a mathematical function depending on the flow intensity and α is a coefficient that depends exclusively on the type of flow. For example, for a shear flow, α is equal to 1/3π. Therefore, Pe progressively grows during the spin-coating process. Equation 2 implies that, when Pe is ‘small’, the intercolloidal forces dominate, thus, *k* ≈ exp­(−*U*_m_/(*k*_B_ *T*)), whereas, when Pe is ‘large’, the flow dominates, thus, *k* ≈ exp­(αPe). The critical Pe value that separates the two regimes is Pe* = *U*_m_/(α*k*_B_ *T*). This means that, by manipulating Pe*, one can change the relative importance of the two regimes during the spin-coating process, thereby anticipating or delaying the crystallization of the material. A possible way to manipulate Pe* is by introducing additives that affect the aggregation barrier, *U*_m_.

The perovskite composition considered in this work is formamidinium tin iodide, FASnI_3_. The effect of additives on film formation was analyzed by preparing three suspensions: a control sample containing formamidinium iodide (FAI, >99.99%, Great Cell) and tin­(II) iodide (SnI_2_, AnhydroBeads, >99.99%, Sigma-Aldrich) dissolved in *N*,*N*-dimethylformamide (DMF, anhydrous, 99.8%, Sigma-Aldrich) at 0.8 M, a sample with 5 mol % tin­(II) chloride (SnCl_2_, >97%, TCI), and a sample with 4-*tert*-butylpyridine (tBP, 98%, Sigma-Aldrich) at a perovskite-to-additive volume ratio of 1.6.^31,32^ After overnight stirring, the suspensions were spin-coated onto indium tin oxide (ITO) coated glass substrates at 2000 rpm in a nitrogen atmosphere to prevent oxidation. The transition from suspension to solid film was studied by *in situ* grazing-incidence wide-angle X-ray scattering (GIWAXS)^33^ and UV–visible (UV–vis) spectroscopy.^34^ In this paper, all *in situ* measurements refer to spin-coating. The scattering measurements were performed at Beamline 12.3.2 at the Advanced Light Source (ALS) of Lawrence Berkeley National Laboratory (LBNL) in the US. During the GIWAXS analysis, an X-ray beam was directed onto the sample at an incidence angle of 2°, while the sample-to-detector configuration maintained a 40° angle. The diffraction data were captured with an exposure time of 1 s using a 2D DECTRIS Pilatus 1 M detector. The UV–vis measurements were performed within a glovebox located at the Molecular Foundry of LBNL. For such measurements, transmission data was collected by using a fiber-coupled Ocean Optics spectrometer (Flame), and each spectrum was acquired with an integration time of 0.1 s. We can estimate the absorbance from the transmittance as −log_10_(*T*). In addition, scanning electron microscopy (SEM) images of the films were taken at the Institute for Applied Sciences and Intelligent Systems of the Italian National Research Council (CNR-ISASI) in Pozzuoli after heating the samples at 140 °C for 20 min in the glovebox environment.

The additives have significant effects on both the properties and the crystallization mechanisms of the perovskite suspensions. Their impact on particle size distribution (PSD) was investigated by dynamic light scattering (DLS): the PSD of the control sample is bimodal; upon addition of SnCl_2_, it becomes monomodal and an enhanced tendency to particle aggregation is observed. When tBP is added, the PSD keeps bimodal, yet a decrease in particle mobility is seen (further details and discussion are provided in the Supporting Information).^35,36^

The *in situ* GIWAXS contour maps shown in Figure 2a–c illustrate the additives’ effect on the perovskite films’ crystallization behavior. In the control sample (see panel a), the diffraction peaks corresponding to the formation of crystalline planes appear after about 40 s. In the sample with SnCl_2_ (panel b), those peaks appear slightly earlier, after about 35 s. On the other hand, when tBP is added (panel c), there is a significant delay in crystallization, with the peaks appearing after about 100 s. Addition of salt to a colloidal suspension reduces the thickness of the EDL around the colloidal particles (the ‘Debye screening length’ ^22,37–39^ ), as it is inversely proportional to the ionic strength of the solution. In turn, a decrease in EDL thickness results in diminished electrostatic repulsion among colloidal particles. According to the classical DLVO theory, such reduction lowers the total interaction energy barrier to aggregation, *U*_m_ in eq 2. Consequently, as ${\mathrm{Pe}}_{{SnCl}_{2}}^{*}<{\mathrm{Pe}}_{Control}^{*}$ , addition of salt shifts the transition between the regime controlled by colloidal interactions and the flow-controlled regime at earlier times with respect to the control case, therefore accelerating the crystallization process. Unlike the effect observed with SnCl_2_, the addition of tBP significantly delays the crystallization process. The tBP molecule contains a nitrogen atom that acts as a Lewis base. This allows the tBP molecule to interact with tin in the nanocrystalline colloids and effectively substitute for iodine.^32,40^ Such substitution reduces the number of available attachment sites, thereby impeding material growth and slowing crystallization kinetics. In this context, the extended DLVO (XDLVO) framework is more appropriate than the classical DLVO theory because the latter only accounts for van der Waals attractions and electrostatic repulsions.^41^ XDLVO, on the other hand, incorporates Lewis acid–base interactions, which may be either attractive or repulsive. However, in our system, tBP binding to the nanocrystalline colloids effectively passivates potential bonding sites, introducing an additional repulsive contribution. Hence, the overall energy barrier to aggregation, *U*_m_, increases. Consequently, the critical Péclet number increases in the presence of tBP $\left(\mathrm{i.e.},{\mathrm{Pe}}_{\mathrm{tBP}}^{*}>{\mathrm{Pe}}_{\mathrm{Control}}^{*}\right)$ , and the colloidal interaction range becomes wider than in the control case, so slowing down the crystallization process. It is noteworthy that there is a a substantial variation (order of magnitude 3) in the crystallization time between SnCl_2_ and tBP that underscores the differences between the effects of the two additives mentioned above.

**Figure 2:**
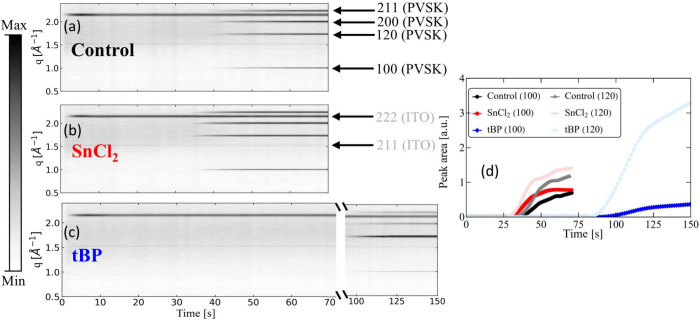
Left: *In situ* GIWAXS contour maps for the control sample (a), SnCl_2_ sample (b), and tBP sample (c). The peaks shown on the plots refer to the ITO and perovskite (PVSK) crystalline planes. Right: Integrated peak area of the 100 and 120 crystalline planes (d) for the three different samples. The normalization is performed by dividing each area by the area of the 222 ITO peak.

Figure 2d shows the time evolution of the peak areas corresponding to the (100) and (120) crystalline planes. This parameter is critical, as it is directly related to the degree of crystallinity along those planes. For both, the (100) and (120) planes, the peak areas of the control and the SnCl_2_ samples grow over time, with the peak area of the SnCl_2_ sample always staying slightly above that of the control sample. On the other hand, when tBP is added, the peak area corresponding to the (100) plane grows less compared to the control sample, whereas the area corresponding to the (120) plane increases more than in the case with SnCl_2_. This suggests that, in the tBP sample, the crystallinity is influenced by the crystallographic orientation, indicating that such additive can also induce changes in the material microstructure (see Supporting Information).

UV–vis spectroscopy is used to investigate the optical properties of the perovskite films in the UV–vis range. *In situ* UV–vis contour maps during spin-coating are reported in Figure 3. The sample containing SnCl_2_ (panel b) shows no significant differences compared to the control case (panel a). The maximum absorbance occurs between 300 and 400 nm and decreases when the film undergoes phase transition during spin-coating, i.e., after about 20 s. At the end of the process, the average absorbance is low, suggesting that SnCl_2_ does not significantly alter the optical properties of the material (for further information, see Supporting Information). It is important to note that the timing between the GIWAXS and UV–vis measurements differs because the experiments were performed under similar, yet not identical, environmental conditions. In particular, GIWAXS was performed in a custom-built nitrogen-filled chamber at the synchrotron end station, whereas UV–vis spectroscopy was carried out in an inert glovebox atmosphere. The evolution of the absorption spectrum of the sample with tBP is markedly different (Figure 3c). Initially, the absorption behavior is the same as in the other two cases, with a peak between 300 and 400 nm, but, when the film arrests (after about 50 s), clear differences appear. By ‘arrest’, we refer to dynamic arrest, which occurs during the spin-coating process when the motion of colloidal particles or crystalline grains becomes immobilized due to solvent evaporation. Indeed, the average absorbance after the dynamic arrest is significantly different than in the previous cases, showing an absorption peak at about 650 nm. Since absorbance is related to the extent and uniformity of material coverage on the substrate, the higher absorbance in the tBP-containing film suggests a more continuous and finely distributed grain morphology than the control and SnCl_2_ samples, which are transparent (see insets in Figure 3).

**Figure 3:**
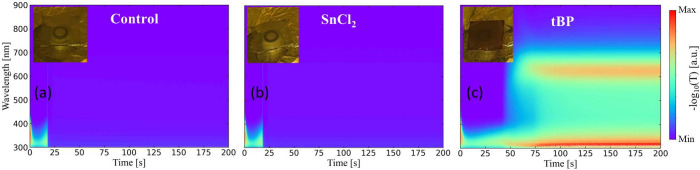
*In situ* UV–vis contour maps for the control sample (a), SnCl_2_ sample (b), and tBP sample (c) during spin-coating. The insets show how the films appear after spin-coating.

The optical properties of the material are intrinsically linked to its microstructural characteristics. An illustration of the microstructure changes induced by the addition of tBP is given in Figure 4. In the control sample (top row), all the attachment sites on the colloidal particles are initially available. During the spin-coating process, the evaporation of the solvent increases the concentration of the colloids, forming clusters that grow until a state of dynamic arrest is reached. For both the control and the SnCl_2_ samples, the arrested state results in crystalline grains of different sizes that are poorly connected, as indicated by the SEM image on the top right (see Supporting Information). This happens because there is no orientation control during colloid attachment, thus grain growth is irregular, yielding a mixture of large grains and poorly bonded smaller grains. In contrast, the presence of tBP leads to a very different scenario (bottom row in Figure 4). Indeed, the additive molecules occupy some sites on the colloidal particles (not adjacent for steric reasons), thus acting as a molecular lubricant that promotes the growth in an oriented manner: as a consequence, when the material reaches the dynamic arrested state, its structure is characterized by a dense network of colloidal aggregates, similar to a chemical gel. The presence of intermediate pyridine-tin complexes is responsible for the absorption peak at 650 nm in Figure 3c, which subsequently disappears during the annealing step due to the volatilization of tBP (see Supporting Information).^40,42,43^ On the other hand, the gel-like structure characterizing the dynamic arrested state results in the formation of a much more compact film with uniform grain distribution, as shown by the SEM image on the bottom right of Figure 4. Since the total amount of perovskite material is the same across all samples, the observed differences in film morphology can be attributed to variations in grain size and distribution. In the control sample, the grains exhibit larger lateral dimensions and height, indicative of less uniform growth. In contrast, the sample containing tBP shows a significantly higher density of grains that are smaller in size and reduced in height, yet more homogeneously distributed across the substrate. Improving these fundamental properties also increases efficiency (see the Supporting Information for more details).

**Figure 4:**
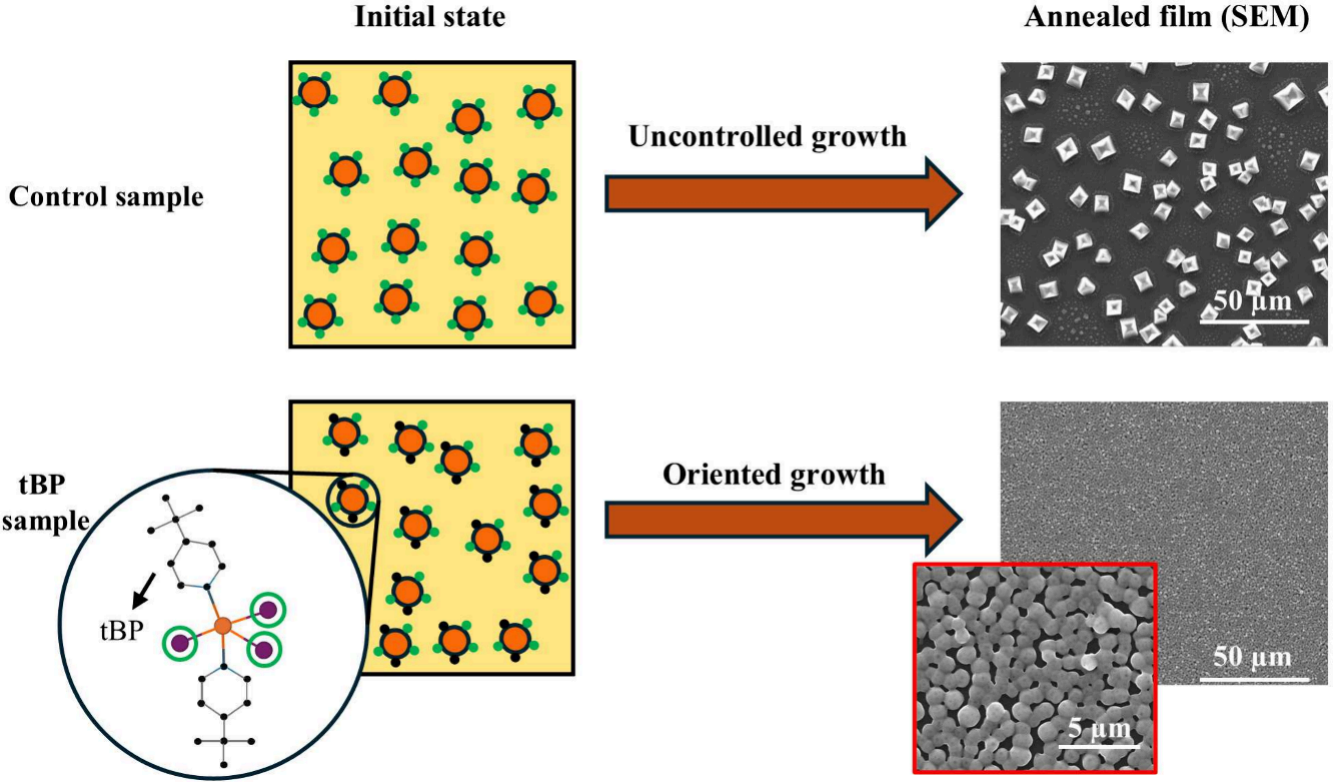
Schematic description of the control sample (top left) and the sample with tBP (bottom left) at the beginning of the spin-coating process (initial state). The orange circles represent the perovskite colloids, the green circles the sites available for the attachment of other colloids, and the black circles the unavailable sites, i.e., those occupied by tBP molecules. The SEM images on the right are taken after sample annealing at 140 °C.

Our approach correlates the colloidal properties of perovskite precursor suspensions with their crystallization dynamics by introducing Pe* as a governing parameter. Specifically, in the case of SnCl_2_, a decrease in Pe* is observed, which is attributed to the reduction in electrostatic repulsive interactions resulting from the thinning of the EDL. Conversely, adding tBP increases Pe*, as this additive forms stable Lewis acid–base complexes with tin in the colloids, hindering particle aggregation. Additionally, we demonstrate that tBP modulates the perovskite growth process by promoting an oriented crystallization, resulting in films with enhanced morphological homogeneity. The positive influence of tBP and other pyridine-based derivatives on the microstructural quality and photovoltaic performance of tin-based perovskite solar cells has been consistently documented in scientific literature.^44–47^

Several additives that play roles similar to those of SnCl_2_ and tBP have been reported for metal halide perovskites. For example, tin fluoride, SnF_2_ promotes the formation of larger aggregates in suspension while providing oxidation suppression for tin and defect passivation.^31^ Similarly, methylammoniumthiocyanate (MASCN) increases perovskite aggregate size and accelerates crystallization upon antisolvent addition.^48^ Among tBP-like additives, dimethyl sulfoxide (DMSO), due to its strong Lewis basicity, forms stable complexes with perovskite precursors. This alters suspension properties and crystallization dynamics.^49,50^ At high concentrations, DMSO induces a gel-like phase that broadens the antisolvent processing window. Comparable effects have been observed with *N*-cyclohexyl-2-pyrrolidone (CHP) in lead-based systems^51^ and with hexamethylphosphoramide (HMPA) in tin-based perovskites.^52^

In this Letter, we propose an entirely novel understanding of the crystallization of perovskite films from precursor suspensions. Our findings demonstrate that modulating Pe* can lead to significant variations in crystallization dynamics and that the colloidal properties of the precursor suspensions are critical in determining the microstructure of the resulting perovskite films. By coupling colloidal interaction theory with the fluid dynamics of the spin-coating process, this approach lays the groundwork for developing a predictive model that can quantitatively assess the influence of additives on crystallization behavior. Importantly, this framework is not limited to spin-coating but can be extended to other solution-based deposition techniques.^53^

To our knowledge, this is the first attempt to generalize the influence of chemical and process-related variables on the crystallization mechanism of perovskite suspensions. We believe that this work provides a new perspective on the description of perovskite film formation processes, giving valuable insights for the future development of high-efficiency solar cells.

## Supplementary Material



## Notes

Additional details on the DLS, UV–vis, and GIWAXS measurements discussed in the main text (PDF)
