# Supplementary material for: Aggregation Dynamics of Colloidal Particles in Tin Perovskite Crystalline Film Formation
Source: ACS Energy Lett. 2025 Oct 28;10(11):5781–7. doi: 10.1021/acsenergylett.5c02847 (PMC12624835; doi:10.1021/acsenergylett.5c02847)
Supplement: Supplementary file 1 [file nz5c02847_si_001.pdf]

Supporting Information: Aggregation dynamics of colloidal particles in tin perovskite crystalline film formation

Davide Amoroso,† Giuseppe Nasti,‡ Massimiliano Maria Villone,† Tim Kodalle,¶ Carolin Maria Sutter-Fella,¶ Pier Luca Maffettone,† and Antonio Abate*,†

†*Department of Chemical, Materials and Manufacturing Engineering, University of Naples Federico II, Piazzale Vincenzo Tecchio, 80, 80125, Naples, Italy*

‡*Enea Research Center Portici, Piazzale Enrico Fermi 1, 80055 Portici, Italy*

¶*Molecular Foundry, Lawrence Berkeley National Laboratory, Berkeley, CA 94720, USA*

E-mail: antonio.abate@unina.it

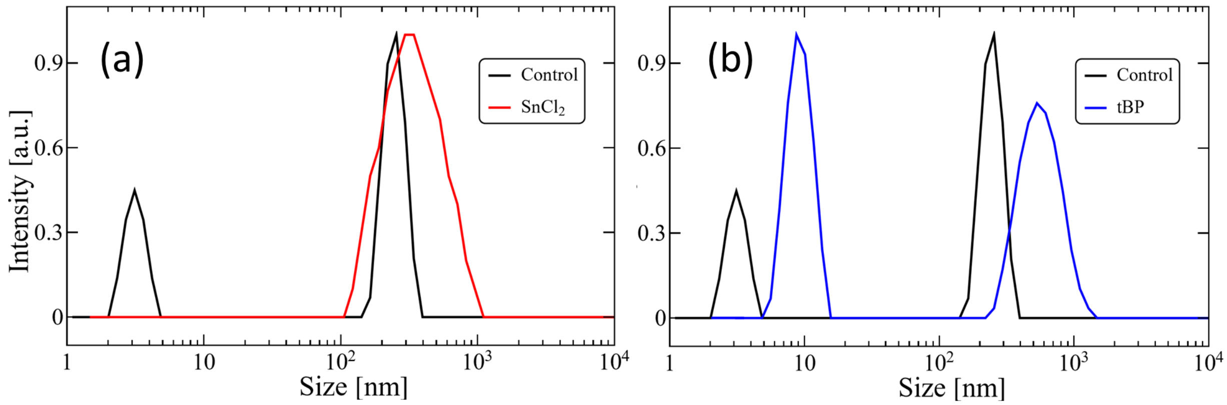

Figure S1: (a) Comparison between the particle size distributions (PSD) of the control sample (black curve) and the $SnCl_2$ sample (red curve) obtained via DLS. (b) Comparison between the PSD of the control sample (black curve) and the tBP sample (blue curve) obtained via DLS.

Figure S1 shows the particle size distributions (PSD), obtained via dynamic light scattering (DLS), of the control perovskite colloidal suspension (black curves in both panels a and b), the sample with $SnCl_2$ (red curve in panel a), and the sample with tBP (blue curve in panel b). The control sample shows a bimodal distribution with peaks centered at approximately 3 nm and 250 nm. Upon addition of $SnCl_2$, the 3 nm peak disappears, whereas the 250 nm peak shifts to 300 nm and becomes broader (see Figure S1a). This behavior can be attributed to the ability of the salt to reduce the electrostatic repulsion among the colloids, thereby promoting aggregation.[1] In contrast, the sample with tBP also shows a bimodal distribution (see blue curve in Figure S1b), but with peaks at larger particle size compared to the control case (specifically, at 9 nm and 550 nm). This shift does not indicate an actual increase in colloid or aggregate size, but is likely due to a decrease in colloid mobility. Indeed, the tBP molecules form Lewis acid-base complexes with the tin colloids, effectively slowing down their motion: since the diffusion coefficient is inversely proportional to the particle size, this results in the 'appearance' of larger particles.

DLS measurements were conducted utilizing a Malvern Zetasizer Nano ZS instrument.[2,3] The suspensions were introduced into quartz cuvettes within the glovebox and sealed using Teflon film and Parafilm. Subsequently, the sealed quartz cuvettes were carefully removed from the glovebox and positioned in the instrument for the measurement. The experimental conditions encompassed a temperature of 20°C, an equilibration duration of 60 seconds, and a backward scattering (173°) configuration.

Figure S2 shows the GIWAXS patterns for the control sample and samples with $SnCl_2$ and tBP, evaluated after the formation of the material and, then, after the appearance of the rings (see main text). It can be seen that the frames appear almost identical for the control and $SnCl_2$ samples, but not for the tBP sample. In the latter, the 120 plane intensity appears to be higher in the out-of-plane direction. This further shows that tBP also has an effect on the orientation of growth of the crystalline grains, which $SnCl_2$ has not.

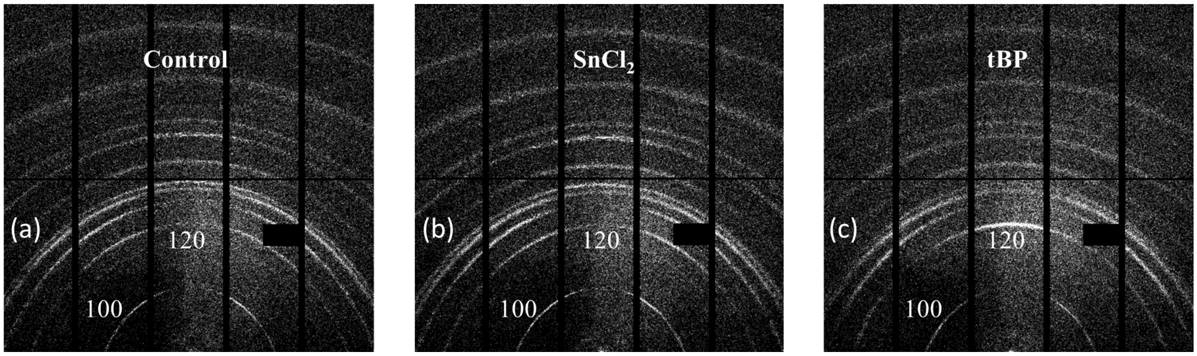

Figure S2: GIWAXS diffraction patterns for the control (a), the $SnCl_2$ and the tBP (c) samples taken at 60 seconds for (a) and (b), and at 110 seconds for (c).

Figure S3 shows the diffraction patterns obtained from the GIWAXS measurements at the conclusion of the spin-coating process for the control, $SnCl_2$, and tBP samples.

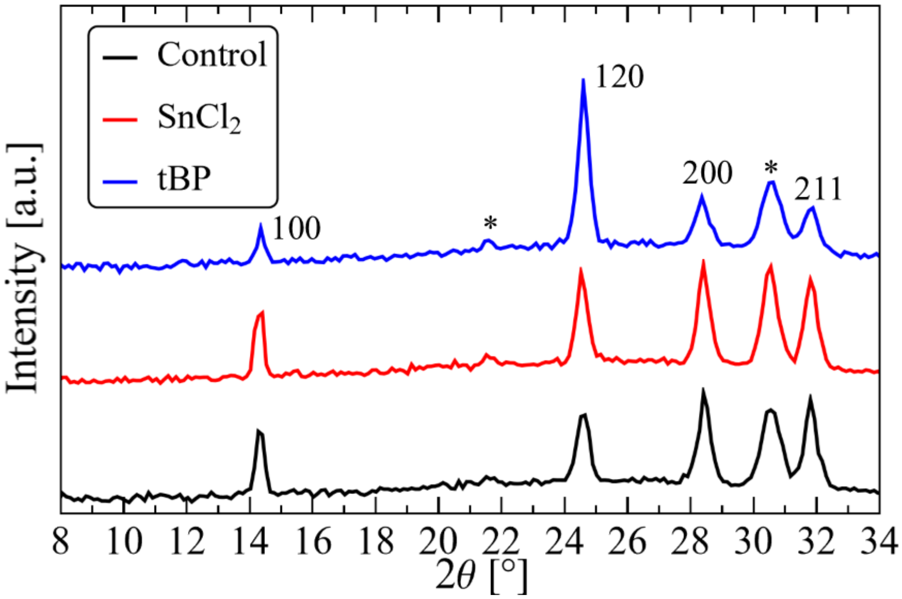

Figure S3: Diffraction patterns of the control (black), $SnCl_2$ (red) and tBP (blue) samples at the end of spin-coating. The asterisks indicate the crystalline planes of the ITO.

The UV-Vis spectra recorded during the spin-coating of the control sample and the sample containing $SnCl_2$ are shown in Figure S4. The data are plotted on a logarithmic scale to emphasize the low absorbance values exhibited by both samples after approximately 20 seconds.

In Figure S5, the UV-Vis spectra during annealing at 140°C for 2 minutes are reported.

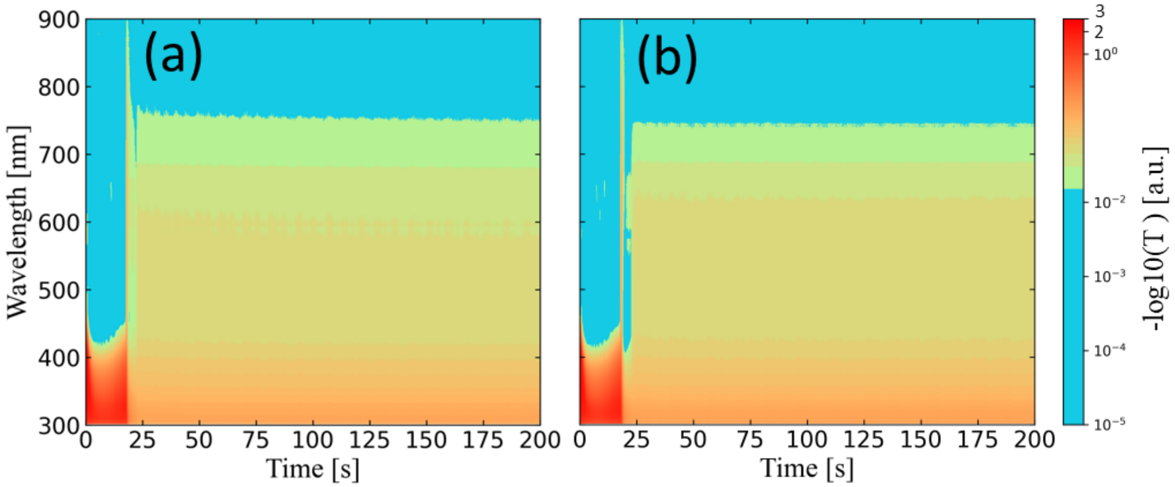

Figure S4: Log-scale UV-Vis contour maps during spin-coating of the control (a) and the $SnCl_2$ (b) samples.

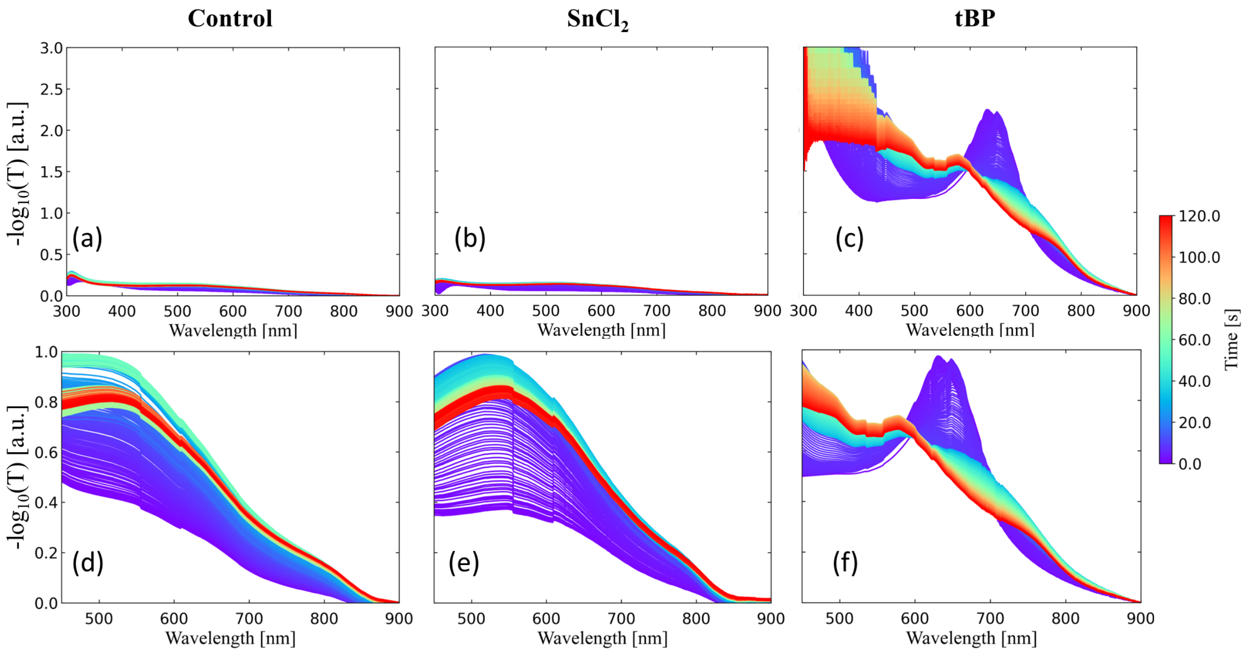

Figure S5: Top: $-\log_{10}(T)$ spectra during annealing for the control (a), $SnCl_2$ (b), and the tBP samples (c). Bottom: normalized spectra of the control (d), $SnCl_2$ (e), and tBP (f) samples between 450 and 900 nm.

For both the control sample (a) and the sample with $SnCl_2$ (b), the spectra show minimal changes and keep almost constant with low absorbance. This suggests that the microstructure undergoes little change during annealing, indicating that an almost complete

crystallization was achieved during the spin-coating process. In contrast, the spectrum of the tBP-containing sample (c) shows the disappearance of the 650 nm peak, evolving into the characteristic absorption spectrum of tin-based perovskites.[4] This spectral change likely reflects a microstructural rearrangement in which the volatilization of tBP occurring at high temperature facilitates the aggregation of colloids in a gel-like structure, thus resulting in the formation of dense, compact crystalline grains. The bottom row of Figure S5 shows normalized plots of the spectra between 450 and 900 nm to emphasize their features.

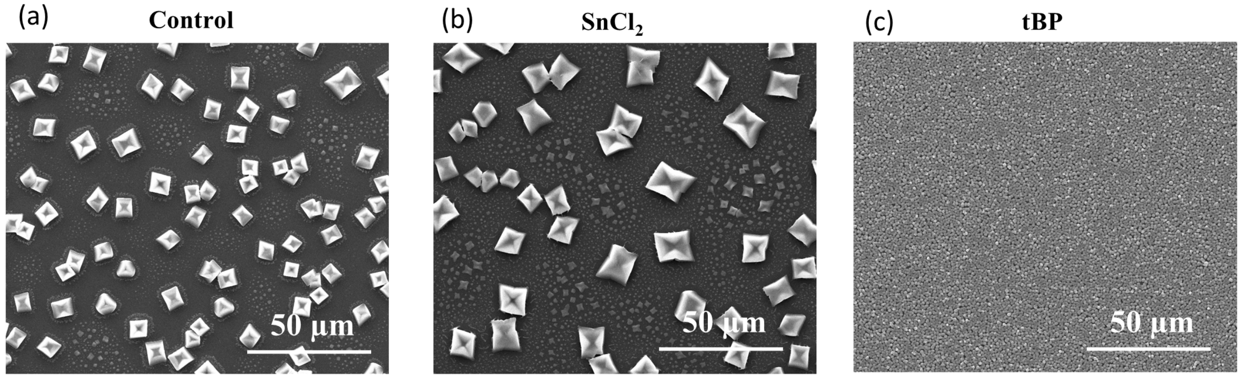

Figure S6: SEM images of the annealed control (a), $SnCl_2$ (b), and tBP (c) samples.

Figure S6 shows SEM images of the samples after thermal annealing at 140 °C. Both the control sample and the sample containing $SnCl_2$ exhibit significant microstructural inhomogeneity, characterized by a broad distribution of grain size and poor inter-grain connectivity. The $SnCl_2$-containing sample shows an increase in average grain size compared to the control sample; anyways, both samples lack uniform coverage and have isolated grains. In contrast, the sample incorporating tBP shows a more uniform and continuous grain distribution, which is indicative of improved crystal growth and film formation.

Figure S7 displays the J–V characteristics of devices that incorporate $SnCl_2$ and tBP as additives. Figure S7a shows how $SnCl_2$ affects $FASnI_3$-based perovskite solar cells, as reported in the referenced study. The addition of $SnCl_2$ yields only a marginal improvement in device performance, with the PCE increasing slightly from 1.15% to 1.28%, compared to

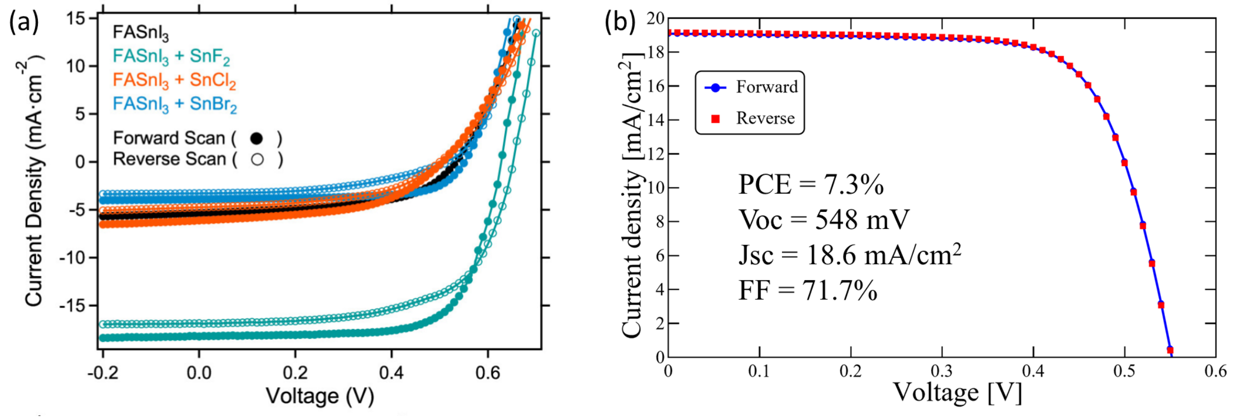

Figure S7: (a) Illuminated current density plot (J-V) for different $FASnI_3$ samples with SnX_2 additives (adapted with permission from Joy et al.[5] Copyright 2024, American Chemical Society). (b) J-V plot for $FASnI_3$ sample with tBP (courtesy of Nasti et al.[6])

the control group without additives. In contrast, Figure S7b shows the J–V curve for the best device prepared with tBP. In this case, all photovoltaic parameters are enhanced, and the device reaches an efficiency of 7.3%. The corresponding control device is not reported because it was nonfunctional.

References

1. Amoroso, D.; Nasti, G.; Sutter-Fella, C. M.; Villone, M. M.; Maffettone, P. L.; Abate, A. The central role of colloids to explain the crystallization dynamics of halide perovskites: A critical review. *Matter* **2024**, *7*, 2399–2430.

2. Hassan, P. A.; Rana, S.; Verma, G. Making Sense of Brownian Motion: Colloid Characterization by Dynamic Light Scattering. *Langmuir* **2015**, *31*, 3–12.

3. Stetefeld, J.; McKenna, S. A.; Patel, T. R. Dynamic light scattering: a practical guide and applications in biomedical sciences. *Biophysical Reviews* **2016**, *8*, 409–427.

4. Wang, C.; Gu, F.; Zhao, Z.; Rao, H.; Qiu, Y.; Cai, Z.; Zhan, G.; Li, X.; Sun, B.; Yu, X.; Zhao, B.; Liu, Z.; Bian, Z.; Huang, C. Self-Repairing Tin-Based Perovskite Solar Cells with a Breakthrough Efficiency Over 11%. *Advanced Materials* **2020**, *32*, 1907623.

5. Joy, S.; Hossain, T.; Tichy, A.; Johnson, S.; Graham, K. R. Defect Modulation via SnX2 Additives in FASnI3 Perovskite Solar Cells. *The Journal of Physical Chemistry Letters* **2024**, *15*, 3851–3858.

6. Nasti, G. et al. Pyridine Controlled Tin Perovskite Crystallization. *ACS Energy Letters* **2022**, *7*, 3197–3203.
